# DNA-assisted selective electrofusion (DASE) of *Escherichia coli* and giant lipid vesicles†

**DOI:** 10.1039/d2nr03105a

**Published:** 2022-08-24

**Authors:** Sho Takamori, Pietro Cicuta, Shoji Takeuchi, Lorenzo Di Michele

**Affiliations:** Department of Physics, University of Cambridge JJ Thomson Avenue Cambridge CB3 0HE UK pc245@cam.ac.uk; Institute of Industrial Science, The University of Tokyo 4-6-1 Komaba Meguro Tokyo 153-8505 Japan takeuchi@hybrid.t.u-tokyo.ac.jp; Artificial Cell Membrane Systems Group, Kanagawa Institute of Industrial Science and Technology 3-2-1 Sakado Takatsu-ku Kawasaki Kanagawa 213-0012 Japan; Department of Mechano-Informatics, Graduate School of Information Science and Technology, The University of Tokyo 7-3-1 Hongo Bunkyo Tokyo 113-8654 Japan; International Research Center for Neurointelligence (IRCN), The University of Tokyo Institutes for Advanced Study (UTIAS), The University of Tokyo 7-3-1 Hongo Bunkyo Tokyo 113-8654 Japan; Department of Chemistry, Imperial College London London W12 0BZ UK l.di-michele@imperial.ac.uk; fabriCELL, Imperial College London London W12 0BZ UK

## Abstract

Synthetic biology and cellular engineering require chemical and physical alterations, which are typically achieved by fusing target cells with each other or with payload-carrying vectors. On one hand, electrofusion can efficiently induce the merging of biological cells and/or synthetic analogues *via* the application of intense DC pulses, but it lacks selectivity and often leads to uncontrolled fusion. On the other hand, synthetic DNA-based constructs, inspired by natural fusogenic proteins, have been shown to induce a selective fusion between membranes, albeit with low efficiency. Here we introduce DNA-assisted selective electrofusion (DASE) which relies on membrane-anchored DNA constructs to bring together the objects one seeks to merge, and applying an electric impulse to trigger their fusion. The DASE process combines the efficiency of standard electrofusion and the selectivity of fusogenic nanostructures, as we demonstrate by inducing and characterizing the fusion of spheroplasts derived from *Escherichia coli* bacteria with cargo-carrying giant lipid vesicles.

## Abstract 2

DNA-assisted selective electrofusion (DASE) combines the efficiency of standard electrofusion with the selectivity of DNA-mediated interactions. Here we apply DASE to induce the fusion between giant lipid vesicles and *E. coli* derived spheroplasts.

## Graphical abstract

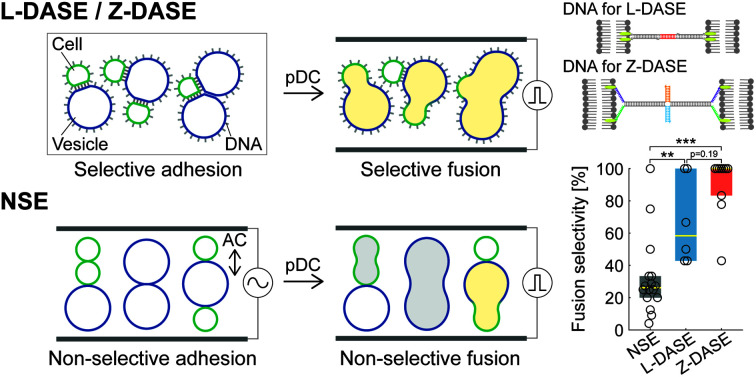

## 1 Introduction

Synthetic biology aspires at re-engineering life forms either to impart functionalities beyond what is observed in nature or simply to help unravel the fundamental workings of life. Accomplishing these tasks often requires significant modification of the genetic, molecular and physical makeup of biological cells.

Genome engineering can be pushed to the extreme with the replacement of the entire bacterial genome with a synthetic version, as achieved with *Mycoplasma mycoides*^1,2^ and *Escherichia coli*,^3,4^ or the partial replacement of eukaryotic chromosomes with *Saccharomyces cerevisiae*.^5–12^ Less radical genome editing has become routine in academic and industrial settings, and more recently in the clinic with the advent of cell therapy.^13^ In all cases, exogenous material needs to be introduced in living cells, be it a brand new genome or the CRISPR–Cas9 machinery required to modify the original genomic DNA.^14–19^ However, while high throughput techniques for the intra-cellular delivery of molecular components are well developed, *e.g.* relying on viral/non-viral transfection methods^20,21^ or electroporation,^22,23^ the direct delivery of bulky components is only feasible with low throughput approaches such as microinjection or, as done for chromosome replacement, by step-wise delivery of short fragments and their *in vivo* assembly.^1,3^

Large-scale cell modification is not restricted to the genome, as successful attempts are being made to equip cells with synthetic organelles hosting enzymatic reactions^24,25^ or enabling the synthesis of non-natural proteins.^26,27^ Pushing this philosophy even further, one can conceive the creation of hybrid cells, built on a natural cell scaffold but featuring an arsenal of artificial parts on their membrane and cytoplasm – a concept that sprouts at the intersection between “conventional” synthetic biology^28^ and the renewed effort of creating artificial cells from the bottom-up.^29–38^

A promising route for the creation of radically engineered hybrid cells would be that of inducing the fusion of the target-cell plasma membrane with a micron-scale carrier, such as a giant lipid vesicle.^39^ Membrane fusion is central to several biological processes including exocytosis,^40^ morphogenesis,^41^ embryogenesis^42^ and fertilization,^43^ and can be mediated by dedicated machinery such as SNARE proteins.^44–46^ Synthetically, membrane fusion can be induced by the application of electric fields (electrofusion),^22,39,47–52^ the addition of polymer depletants,^51,53^ the local heating action of nanoparticles,^54,55^ or the electrostatic attraction between lipid head groups.^56,57^ While these approaches afford good efficiency and throughput, they lack the selectivity of biological machinery.

Inspired by the structure and action of SNARE proteins, the tools of DNA nanotechnology have been applied to construct artificial fusogenic nanomachines^58,59^ from lipid-conjugated nucleic acids^60–68^ or peptide nucleic acids.^69–71^ However, despite their ability to selectively and controllably induce the fusion of membranes, fusogenic DNA nanodevices display relatively low content mixing efficiencies, particularly at room or physiological temperature,^65,66,69^ ascribable to multiple factors including limited affinity of the fusogenic constructs for the membranes or steric repulsion induced by the construct themselves.

Here we introduce DNA-Assisted Selective Electrofusion (DASE), combining the efficiency and high throughput of standard membrane electrofusion with the selectivity of DNA-based fusogenic nanostructures.

**Fig. 1:**
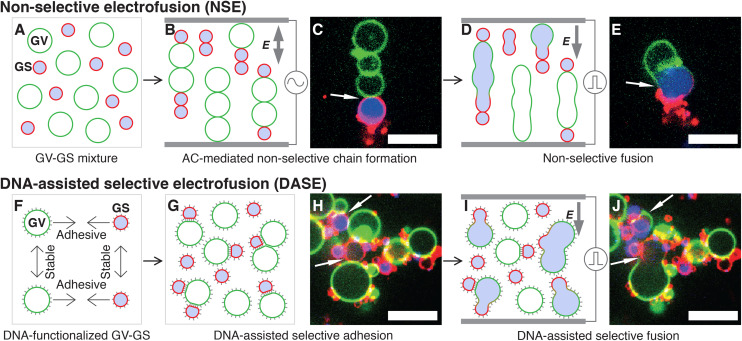
Comparing standard Non-Selective Electrofusion (NSE) and the new DNA-Assisted Selective Electrofusion (DASE) on a suspension of *E. coli* Giant Spheroplast (GS) and Giant lipid Vesicles (GV). (A) In NSE, GSs and GVs are prepared separately and mixed. (B and C) An AC electric field is applied, leading to the formation of non-specific pearl-chain-like aggregates of GSs and GVs. (D and E) A DC pulse is applied inducing fusion between neighboring elements in the chains. Due to non-selective chain formation, fusion also occurs non-selectively. (F) In DASE, GSs and GVs are functionalized with membrane-anchored DNA leading to selective GS–GV adhesion mediated by Watson–Crick base pairing, and steric repulsion between GV–GV and GS–GS, whose DNA coatings are non-complementary. (G and H) The functionalized GSs and GVs are mixed, and the sample is incubated to allow for sufficient selective GS–GV contacts to form. (I and J) The application of the DC pulse leads to selective GS–GV fusion. In the representative confocal micrographs (C, E, H and J), the membranes of GS and GV are stained with TopFluor cholesterol (green) and FM4-64 (red) respectively, while the nucleoid within GS is stained with SYTO41 (blue). Scale bars: 10 μm. Movies of the events in panels C + E and H + J are shown in ESI Videos 1 and 9,† respectively.

In Non-Selective (standard) Electrofusion (NSE), the target cells or lipid vesicles are brought in close proximity by an AC electric field, leading to their alignment in chain-like assemblies (Fig. 1A–C), followed by the application of an intense DC pulse that prompts membrane fusion (Fig. 1D and E). The binding action of the AC field is however non-selective, and one cannot force fusion to occur only between specific objects in a mixed sample. In other words, if the aim is that of fusing cells with synthetic vesicles, NSE would just as easily cause cell–cell and vesicle–vesicle fusion, reducing yield and generating undesired by-products.

In DASE we replace the action of the AC field with membrane-anchored DNA nanostructures, which thanks to the selectivity of Watson–Crick base pairing only connect the objects that we aim to fuse (Fig. 1F–H), before applying the DC pulse to trigger fusion (Fig. 1I and J). Two types of DNA constructs are tested, Linkers that connect the target objects while keeping their membranes a few nanometers apart, separated by a DNA brush,^59,72,73^ and Zippers, which are modelled after the previously reported fusogenic motifs^60–68^ with the aim of bringing the membranes in molecular proximity and aiding the DC-induced fusion.

We test DASE by demonstrating the fusion between *E. coli* Giant Spheroplasts (GS) and cargo-bearing Giant lipid Vesicles (GV) finding that, compared to NSE, it affords a higher selectivity by boosting the ratio of sought (GS–GV) over unwanted (GS–GS and GV–GV) fusion events. Additionally, DASE yields an overall greater throughput by enabling the use of higher cell/vesicle concentrations compared to NSE.

We argue that, thanks to its high throughput and efficiency, DASE could be a valuable tool in the arsenal of modern synthetic biology, facilitating the production of hybrid cells embedding many exogenous components in their cytoplasm and membranes. Additionally, the technology could improve the outcome of genome-editing protocols requiring the efficient intra-cellular delivery of editing machinery and genetic material in research and clinical settings.

## 2 Results and discussion

### 2.1 Formation of *E. coli* giant spheroplast

**Fig. 2:**
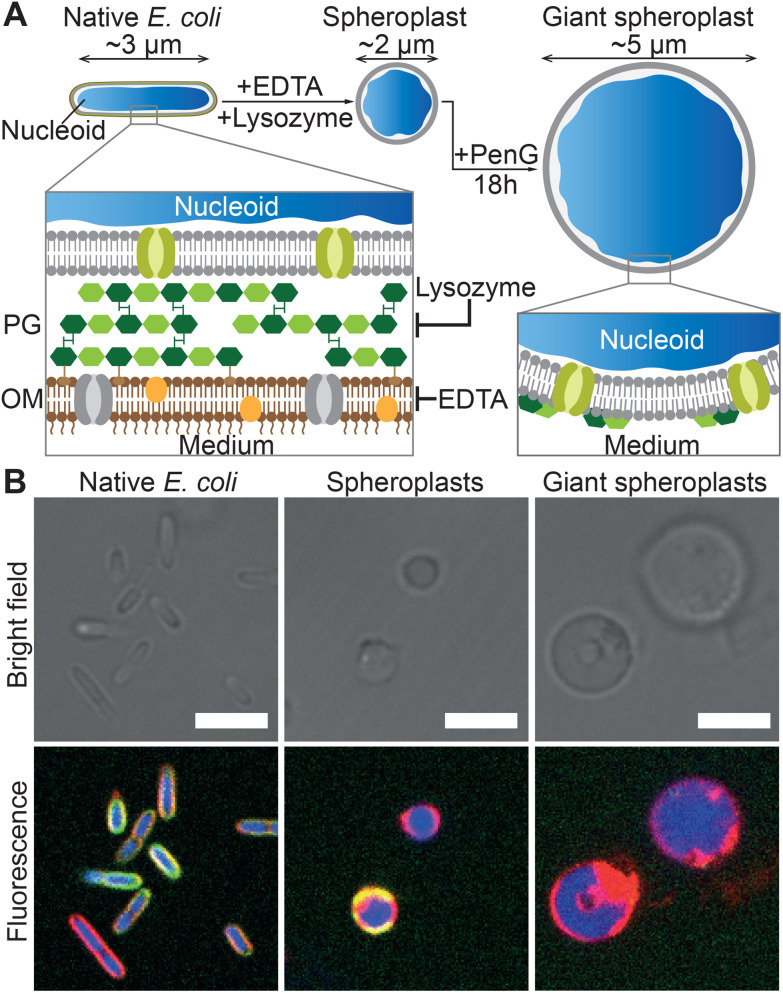
Preparation and structure of *E. coli* giant spheroplasts. (A) Schematic of the protocol for the preparation of Giant Spheroplasts (GSs). Native *E. coli* are incubated with EDTA and lysozyme to remove the outer lipid membrane (OM) and peptidoglycan (PG) cell wall, leading to the formation of spherical spheroplasts. These are then incubated with penicillin G (PenG) to inhibit regeneration of the peptidoglycan and induce their growth to GSs with typical diameters exceeding 5 μm. (B) Confocal micrographs of *E. coli* HST08 cells in the three stages in panel A. The cytoplasmic membrane was stained with FM4-64 (red), the peptidoglycan with WGA–AF488 (green) and the nucleoid with SYTO41 (blue). Scale bars: 5 μm. Radial fluorescence profiles of the three stages are shown in Fig. S1.†

As summarized in Fig. 2A, GSs were prepared from native *E. coli* by stripping away their outer membrane and rigid cell wall, which causes the bacteria to relax from their usual sphero-cylindrical shape to spheres with diameter close to 2 μm. Afterwards, the so-formed spheroplasts were further incubated with an antibiotic to prevent cell wall regeneration and induce their growth to typical sizes in excess of 5 μm, hence becoming giant spheroplasts. Confocal micrographs of native *E. coli* HST08, the spheroplasts and the GSs (Fig. 2B), and the radially-averaged intensity profiles (Fig. S1†) prove that while the *E. coli* nucleoid (blue, SYTO41) and cytoplasmic membrane (red, FM4-64) are retained, the signal from the cell wall (green, WGA–AF488) is mostly lost in their transition to spheroplast and GS. Full details on GS production are reported in the Methods section.

### 2.2 DNA-construct design and membrane functionalization

**Fig. 3:**
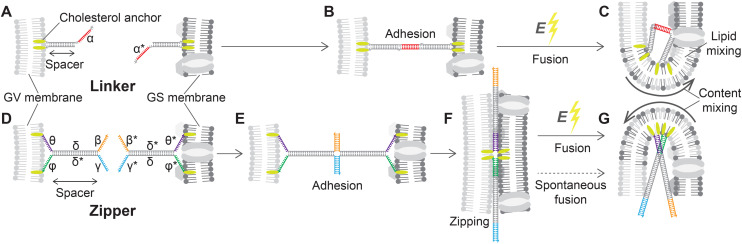
Linker and Zipper DNA constructs induce membrane adhesion and promote fusion. (A) DNA Linkers feature a 15 bp dsDNA spacer. At one extremity, the spacer displays two covalently-attached cholesterol modifications, warranting stable insertion into the lipid membranes,^59^ while on the other side it features a 10 nt ssDNA “sticky end”. Two Linkers are prepared, with complementary sticky ends *α* and α* that selectively bind to each other, and are attached to the surfaces of GVs and GSs, respectively. (B) When the Linkers hybridize they lead to selective adhesion of functionalized GVs and GSs, but the dsDNA spacers provide a degree of entropic repulsion between the linked membranes, which are kept at a distance comparable to the spacer length.^72^ (C) The application of a DC pulse facilitates the fusion of linked membranes. (D) Zippers share a similar architecture to Linkers, with a 21 bp dsDNA spacer and double cholesterol anchor, but each construct features two 12 nt sticky ends *β* and *γ*, complementary to β* and γ* on the other nanostructure. Additionally, 12 nt ssDNA domains are left between the dsDNA spacers and the hydrophobic modifications: *θ* and *ϕ* on one Zipper and their complementary θ* and ϕ* on the second. (E) The two domains making up the spacers of Zippers *δ* and δ* are shared between the two constructs, so that binding initiated by the two pairs of sticky ends progresses through branch migration until the two constructs zip-up fully, bringing the membranes in close proximity (F) and further facilitating fusion when the DC pulse is applied (G). Sequences of all ssDNA components are shown in ESI Table S1.†

The removal of the stiff cell wall makes GSs suitable for electrofusion experiments with Giant lipid Vesicles (GVs), which we prepared from 1,2-dioleoyl-*sn-glycero*-3-phosphocholine (DOPC, see Methods). For DNA-assisted selective electrofusion, GSs and GVs were functionalized with amphiphilic DNA constructs, whose architecture and mode of operation are summarized in Fig. 3. Two types of constructs were designed, dubbed Linkers (Fig. 3A) and Zippers (Fig. 3D). Both nanodevice types feature a double-stranded (ds) DNA spacer, which being relatively short (15 bp for Linkers and 21 bp for Zippers) compared to the persistence length of dsDNA (∼150 bp ^74^) can be regarded as rigid. At one end of the spacer, both DNA termini are covalently modified with cholesterol moieties, rendering the constructs amphiphilic and enabling their stable insertion in both synthetic and biological lipid membranes.^59,75,76^ Note that in fluid membranes, cholesterol-anchored constructs are able to diffuse laterally.^59^ Single-stranded (ss) DNA domains, dubbed sticky ends, are present on the opposite side of the spacer; one for Linkers and two for Zippers (Fig. 3). The latter feature additional unpaired ssDNA domains between the cholesterol anchors and the dsDNA spacer (Fig. 3D).

Two different types of Linkers are prepared, featuring a 10 nt sticky end of sequence *α* or its complementary α* (Fig. 3A). As extensively demonstrated, membranes decorated with complementary Linkers readily adhere to each other.^58,59,72,73,77^ However, the rigid dsDNA spacers form a dense brush in the contact area between adhering membranes, keeping them at a distance comparable with the spacer length^78^ and hence preventing molecular contact between the lipids and spontaneous fusion (Fig. 3B).

Similarly to Linkers, also Zippers are designed as two mutually complementary versions, one with 12 nt sticky ends *β* and *γ* and the other with complementary domains β* and γ* (Fig. 3D). Differently from Linkers, however, the sticky ends of Zippers are oriented parallel to their complementary, such that Zipper–Zipper hybridization brings the membranes closer (Fig. 3E). Furthermore, the domain sequences of the spacers (*δ* and δ*) are identical for both Zippers, so that the zipping action initiated by the sticky ends can propagate through the spacers *via* (four-way) branch migration. Also the 12 nt ssDNA domains separating the spacers from the cholesterol anchors are mutually complementary between the two Zipper-types (*θ*–θ* and *ϕ*–ϕ* in Fig. 3D) thus further facilitating zipping until each of the two strands initially forming one Zipper is fully hybridized to its complementary from the second Zipper, bringing the adhering membranes in molecular proximity (Fig. 3F). The process is thermodynamically driven by the drastically higher number of base-pairing bonds formed in the final (90 bp) compared to the initial configuration (42 bp), and with architectures similar to the ones utilized here it has been demonstrated to promote hemi-fusion and, albeit less efficiently, complete fusion between membranes.^60–62,66,79^

**Fig. 4:**
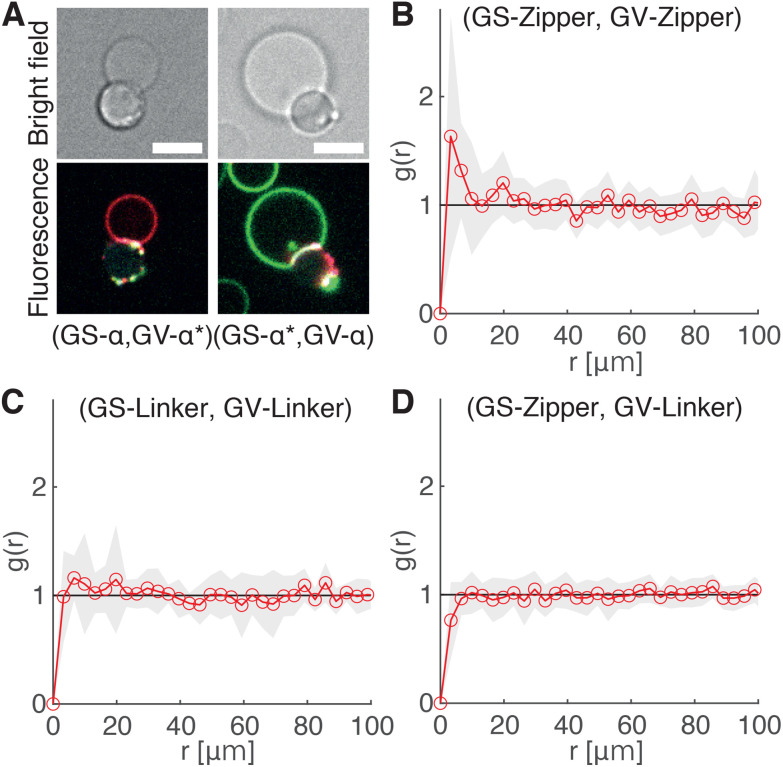
DNA Linkers and Zippers induce selective GS–GV adhesion. (A) Confocal (bottom) and bright field (top) micrographs of interacting GSs and GVs functionalized respectively with Linkers *α*/α* (left) and α*/*α* (right). Here Linkers *α* and α* are respectively labelled with ATTO488 (green) and ATTO590 (red), which can FRET when Linker–Linker bonds are formed. The FRET signal, shown in blue, overlaps with the red and green signals from donor and acceptor at the GS–GV interface, which appears white as a result. Scale bars: 5 μm. (B–D) Radial pair-distribution function (*g*(*r*)) of GS and GV positions in samples where GSs and GVs are functionalized with complementary Zippers (B) complementary Linkers (C) and non-complementary Zipper (GS) and Linker (GV) constructs (D). While *g*(*r*) in B and C show a maximum at short separations, indicative of GS–GV adhesion, the curve is smooth and ∼1 in D, indicative of a random distribution. See also Fig. S2† for the case of GSs and GVs decorated with non-complementary Linkers and Zippers, respectively.

The ability of the cholesterolized DNA constructs to bind GS and GV membranes is proven in Fig. 4A using fluorescently labelled Linkers, which also visually demonstrates GS–GV adhesion mediated by complementary Linkers. The selective adhesion induced by both Zippers and Linkers is quantified by evaluating the radial pair correlation function of the GS and GV positions, highlighting the relative likelihood of GS–GV pairs to be found at a certain distance (Fig. 4B–D and Methods). The more pronounced peak at close GS–GV separations found for Zippers (Fig. 4B) highlights their greater ability to induce GS–GV adhesion compared to Linkers (Fig. 4C), which may follow from the overall stronger Zipper–Zipper hybridization free energy. Note that differently from the previously studied cases in which both the functionalized membranes are synthetic, here DNA-mediated attraction is likely disrupted by the presence of membrane proteins and possibly leftover outer membrane and peptidoglycan on the GS surface, which may “bury” the DNA constructs and/or produce steric repulsion. The latter may be the source of the (fluorescent) aggregates observed on the GS membranes (*e.g.*Fig. 4A). Pair-correlation measurement on samples in which GSs and GVs are functionalized with non-complementary Zippers and Linkers, respectively, shows no evidence of clustering, as expected given that non-sticky DNA constructs are known to provide steric stabilization and thus can help preventing non-specific membrane–membrane adhesion^80^ (Fig. 4D and Fig. S2†). This feature is particularly desirable in the context of DASE, as it could improve selectivity.

### 2.3 Non-selective and DNA-assisted selective electrofusion of GSs and GVs

**Fig. 5:**
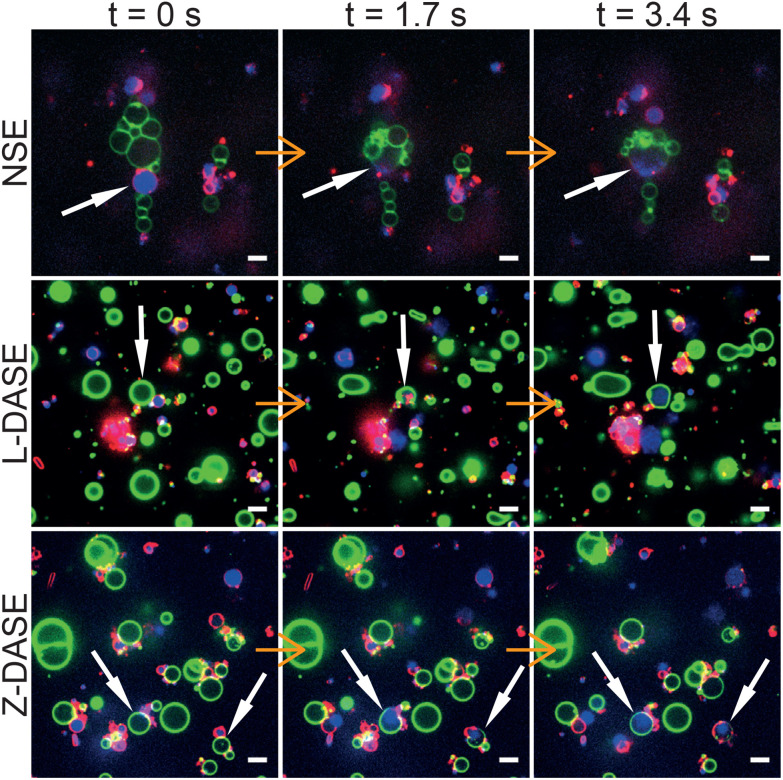
Confocal microscopy demonstrates non-selective and DNA-assisted selective electrofusion of GSs and GVs. In NSE, an AC field (1.9 MHz and 4 V_RMS_) was applied leading the formation of non-specific chain-like aggregates of GSs and GVs. For L-DASE and Z-DASE, DNA-functionalized GSs and GVs were mixed and incubated for 2 hours prior to the experiment. The formation of adhering GS–GV pairs is visible. At *t* = 0 s, a DC pulse (50 μs width and 600 V mm^−1^) was applied to induce fusion, which in all cases occurred by *t* = 1.7 s. By *t* = 3.4 s, spreading of the GS nucleoid into the cytoplasm of the newly formed hybrid cell is visible, as is the mixing of the GS and GV membranes. Relaxation of the fused membranes into more spherical shapes is also visible by comparing frames at *t* = 1.7 s and *t* = 3.4 s. GSs were stained with FM4-64 (red, cytoplasmic membrane) and SYTO41 (blue, nucleoid). GVs were labelled with TopFluor Cholesterol (TFC, green, DOPC/TFC = 98/2 molar ratio). Scale bars: 5 μm. Movies of the events are shown in ESI Videos 2 (NSE), 6 (L-DASE) and 10 (Z-DASE).† See also other cases of NSE, L-DASE and Z-DASE experiments in Fig. S3.†

Fig. 5 shows confocal-image sequences capturing some examples of GS–GV fusion events observed with NSE (top), Linker L-DASE (middle) and Zipper Z-DASE (bottom), while movies from these examples and others are shown in ESI Videos 1–12.† The vertical chain-like aggregates induced by the AC field are clearly visible for the case of NSE, while in DASE less ordered aggregates are formed as a result of DNA-mediated adhesion. Note the clear difference in size between the small DNA-induced clusters and the extended chains produced by the AC field. Being oriented perpendicular to the cell electrodes, the latter are found to cause short circuits if the concentration of GSs and GVs is too high, effectively limiting the throughput of NSE as discussed below. After the DC pulse is applied, merging between the GS cytoplasmic membranes (red, FM4-64) and the GV membranes (green, TopFluor Cholesterol/TFC) is clearly visible in both NSE and DASE, as is the transfer of genetic material (blue, SYTO41) from the GS to the hybrid cell formed after applying the DC pulse. See also another example of GS–GV fusion in each NSE, L-DASE and Z-DASE experiment in Fig. S3.†

**Fig. 6:**
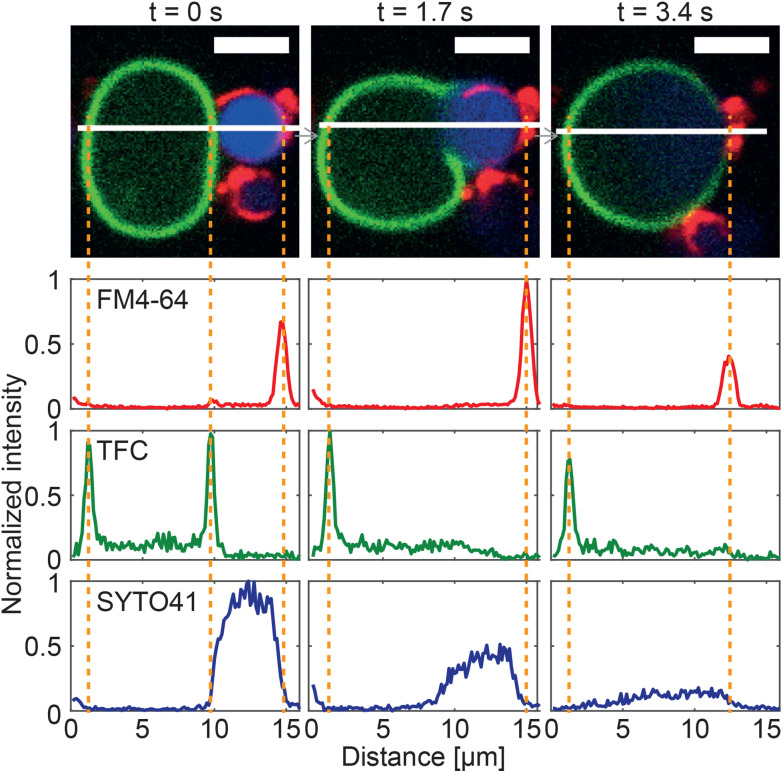
Image cross-sections confirm membrane fusion in a GS–GV NSE experiment. Three consecutive confocal microscopy frames of an NSE event are shown (top), where the membrane and nucleoid of the GS are shown in red (FM4-64) and blue (SYTO41), respectively, and the GV membrane is shown in green (TopFluor Cholesterol/TFC). A DC pulse is applied just after *t* = 0 s (50 μs duration, 600 V mm^−1^ amplitude). Fluorescence intensity cross-sections for the three channels were extracted along the white line. At *t* = 0 s, two peaks from the membranes of the GS and the GV are detectable in both FM4-64 and TFC channels, while the signal from the genetic material of the GS is uniformly distributed in the region between the two GS-membrane peaks. By *t* = 1.7 s membrane fusion has occurred, leading to the disappearance of the GS–GV interface and the associated membrane peaks. A slight initial spread of the GS nucleoid in the lumen of the GV is detected. By *t* = 3.4 s, the SYTO41 signal has spread broadly between the two peaks from FM4-64 and TFC. While an initial mixing of the membrane components is visible in the micrographs, the fluorescent labels are not yet uniformly distributed across the surface of the hybrid cell by this point. Scale bars: 5 μm. See ESI Video 4.†

Fig. 6 reports on the quantitative image analysis of the fusion process. In Fig. 6, we follow the time evolution of the fluorescence intensity of dyes staining the GS cytoplasmic membrane, its nucleoid and the GV membrane (as in Fig. 5) along a cross section which intersects both objects and their interface. Before the application of the DC pulse, peaks corresponding to both membranes are clearly detectable, and the nucleoid of the GS is enclosed by its cytoplasmic membrane. Soon after the application of DC pulse, which occurs between *t* = 0 s and 1.7 s, the GV and GS membrane peaks disappear from the contact region between the two objects, indicating membrane rupture and the onset of fusion, which is also signalled by an initial spreading of the SYTO41 signal in the direction of the GV. At later times, the nucleoid further diffuses into the lumen of the newly formed hybrid cell, which by this stage has already relaxed into a spherical shape. A video of the event in Fig. 6 is shown in ESI Video 4.†

The fusion event highlighted in Fig. 6 is an NSE experiment. Similar trends are observed in both L-DASE and Z-DASE, as demonstrated in Fig. S4, S5, and ESI Videos 6 and 10.† Note also that, to the best of our knowledge, the ones reported here are the first examples of electrofusion, standard or otherwise, involving *E. coli* and synthetic giant lipid vesicles.

**Fig. 7:**
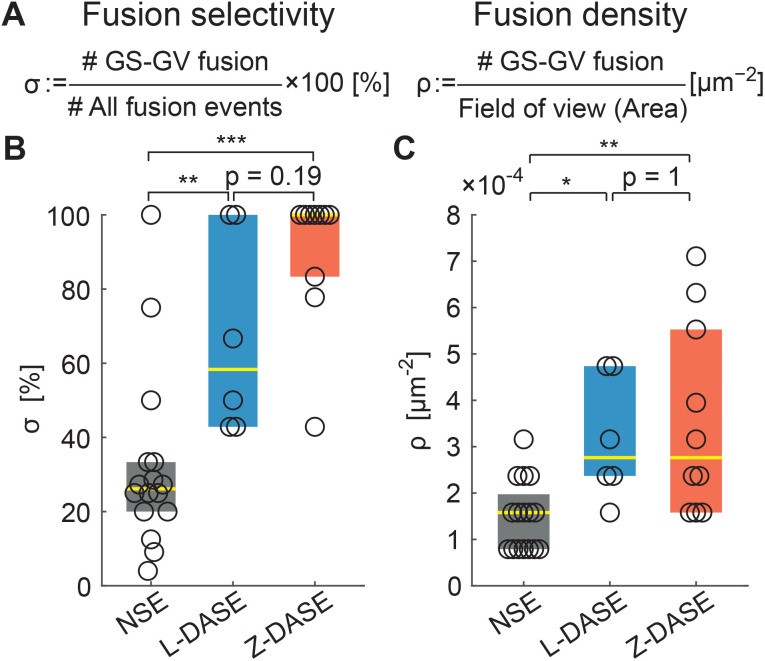
DASE improves fusion selectivity and throughput compared to NSE. (A) The fusion selectivity *σ* is defined as the percentage of specific GS–GV fusion events over all observed events (GS–GV + GS–GS + GV–GV). The fusion (areal) density *ρ* is defined as the number of GS–GV fusion events over the area of the field of view, and used as a measure of throughput. (B) Z-DASE displays the highest median selectivity (100%, *N* = 10 fields of view), followed by L-DASE (58.3%, *N* = 6) and NSE (26.1%, *N* = 16). (C) Z- and L-DASE also improve fusion density, with respective medians of 2.8 × 10^−4^ μm^−2^ (*N* = 10) and 2.8 × 10^−4^ μm^−2^ (*N* = 6) compared with 1.6 × 10^−4^ μm^−2^ (*N* = 16) for NSE. In all box plots, each point indicates one field of view, the yellow line marks the median and the colored (dark grey/blue/red) rectangles mark the 25th and 75th centiles. See Methods for the computation and annotation of *p*-values. Raw data are summarized in Tables S2–S4.†

Having demonstrated the electrofusion of GSs and GVs, both with the traditional approach and by replacing the clustering action of AC with sticky DNA constructs, we proceed with comparing the performance of NSE and DASE, as summarized in Fig. 7. We define the selectivity *σ* as the percentage of specific GS–GV fusion events over the total number of fusions (GS–GV, GS–GS and GV–GV) observed within a microscopy field of view after an NSE or DASE run (Fig. 7A). Data in Fig. 7B demonstrates a significant improvement in selectivity between NSE (*σ* ∼ 26.1%), L-DASE (*σ* ∼ 58.3%), and Z-DASE, which approaches complete selectivity (*σ* ∼ 100%). Generally, the improved selectivity of DASE compared to NSE is easily rationalized as a consequence of the intrinsic lack of specificity of the latter, following from the indiscriminate action of the AC field which brings together random objects in the chain-like aggregates. In turn, the enhanced performance of Z-DASE compared to L-DASE may be a consequence of the ability of Zippers to take membranes in closer proximity compared to Linkers, eliminating the repulsive action of the dsDNA brush that separates adhering membranes for the latter, as graphically demonstrated in Fig. 3. In addition, Zippers are found to generally produce more GS–GV contacts compared to Linkers, as discussed above and shown in Fig. 4B and C.

As a representative measure of throughput we use the areal density of specific fusion events, *ρ*, calculated as the number of observed GS–GV fusions per unit area of a microscopy frame (Fig. 7A). Indeed all GSs and GVs sediment to the bottom of the electrofusion cell, where confocal images are acquired, hence *ρ* is proportional to the total number of sought hybrid cells produced in each run.

Fig. 7C demonstrates that *ρ* is substantially (∼2×) higher in L-DASE and Z-DASE compared to NSE (Fig. 7C), with Linkers and Zippers displaying similar efficiencies.

The improved throughput of DASE compared to NSE derives from the greater GS and GV concentration affordable in the former case, highlighted in Fig. S6A.† Indeed, in NSE the concentration of GSs and GVs cannot be increased to the same values used for DASE, as the formation of the long chain-like aggregates would otherwise short-circuit the electrodes (Fig. 5 NSE. See also Fig. S3† NSE). This issue does not arise in DASE, where more compact (non-percolating) GS–GV clusters form thanks to DNA-mediated interactions (Fig. 5 L-DASE and Z-DASE. See also Fig. S3† L-DASE and Z-DASE).

In fact, as demonstrated in Fig. S6B–D,† the fusion probability calculated as a percentage of the total number of GSs and GVs is higher for NSE than for DASE. Yet, DASE yields more hybrid cells per experiment because a far higher number of GSs and GVs can be processed.

In Tables S2–S5† we summarize the number of observed GSs and GVs, and fusion events in each electrofusion experiment, from which the quantities in Fig. 7 and S6† are extracted.

### 2.4 Intra-cellular delivery of molecular cargos by DASE and spontaneous fusion

**Fig. 8:**
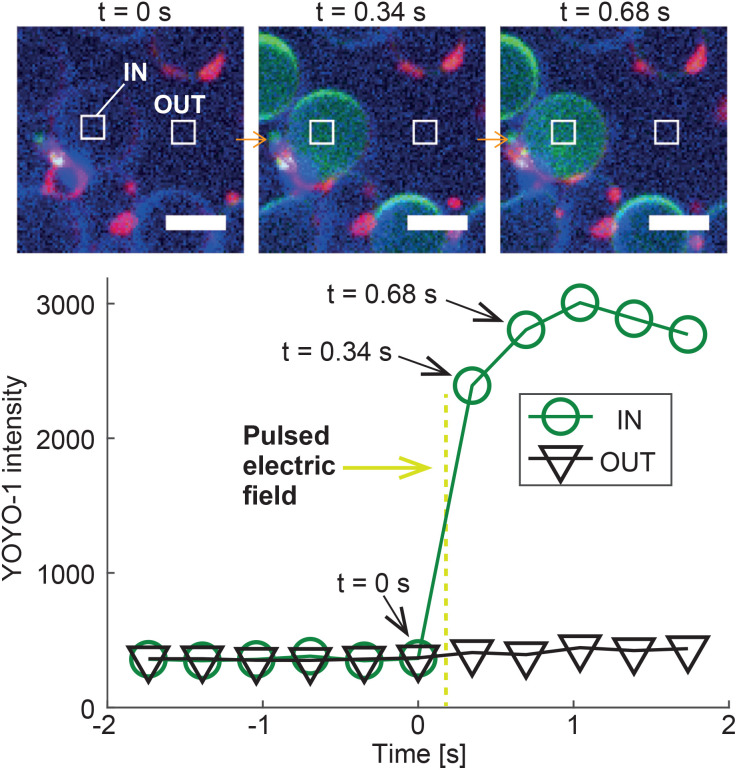
Intra-cellular delivery of GV-encapsulated YOYO-1 into a GS by Z-DASE. The DNA-intercalating dye YOYO-1 is encapsulated in the GVs, whose membranes are stained with ATTO390-DPPE (blue). GSs are only labelled with the membrane stain FM4-64 (red). Prior to the Z-DASE experiment, both GSs and GVs are functionalized with Zippers and mixed to enable adhesion as described in Methods. The YOYO-1 signal (green) is monitored in a region of interest inside the GV (IN) and one in a nearby empty region (OUT), and the average intensity recorded in each region is shown as a function of time. Initially, only background YOYO-1 signal is detected both inside and outside the GV. After the application of the DC pulse between *t* = 0 s and 0.34 s (yellow dashed line, 50 μs width, 600 V mm^−1^ amplitude), the YOYO-1 signal collected within the GV shows a rapid increase before saturating, indicating the fusion and mixing between the intercalating dye and the GS nucleoid in the newly formed hybrid cell. The signal recorded outside the hybrid cell does not change, suggesting that YOYO-1 and/or *E. coli* genetic material did not substantially leak during fusion. The event is shown in ESI Video 12.† Scale bars: 5 μm. Similar trends are observed for L-DASE and NSE, as shown in Fig. S7, S8 and ESI Videos 5 and 8.†

To demonstrate the potential of DASE as a tool for intra-cellular cargo-delivery, we conducted GS–GV DASE and NSE experiments using GVs loaded with DNA intercalating dye YOYO-1, which experiences a significant increase in fluorescence quantum yield when binding double-stranded DNA (Fig. 8 and S7, S8†). Therefore, upon GS–GV fusion, we expect a fluorescent signal emerging in the lumen of the formed hybrid cell, following exposure of the *E. coli* nucleoid to YOYO-1. Fig. 8 shows the outcome of a Z-DASE fusion event. A sequence of confocal images (Fig. 8, top) confirms a sharp increase in YOYO-1 emission immediately after the DC pulse is applied, which can be quantified by sampling the fluorescence intensity within the hybrid cell (region labelled as “IN” in Fig. 8). After the initial increase, the signal plateaus before slightly decreasing, possibly following spreading of the stained nucleic acids within the hybrid cell (Fig. 8, bottom). We can thus confirm the delivery of a model cargo within a GV following fusion with a GS. Conversely, no increase in the YOYO-1 signal was detected in the nearby medium, hinting at the limited DNA/YOYO-1 leakage during fusion (region labelled as “OUT” in Fig. 8). See also a video of the event in ESI Video 12.† Similar trends were observed in L-DASE and NSE experiments using GSs and YOYO-1-encapsulating GVs, as shown in Fig. S7, S8 and ESI Videos 5 and 8.† For these experiments, the GS nucleoid was not stained with SYTO41.

Finally, we note that Zippers are sometimes able to induce spontaneous fusion between GSs and GVs, namely without the application of any DC impulse. These events are rare, but they do occur after samples of Zipper functionalized GSs and GVs are incubated for several hours (see Methods), as exemplified in Fig. S9.† The spontaneous (and complete) membrane fusion induced by Zipper-like constructs is reported to occur with relatively low efficiency in pairs of smaller synthetic vesicles,^62,63,66^ and it has also been seen between small synthetic vesicles and cells.^68^ The low efficiency of spontaneous fusion in our system is ascribable to various factors, including the fact that we do not select highly fusogenic lipid compositions for our GVs (*e.g.* featuring large mole fractions of fusogenic lipids such as DOPE), the large size and negligible local curvature of GVs that makes their bilayer more stable compared with the smaller liposomes used in spontaneous fusion studies (which however cannot encapsulate large cargoes), and possibly steric barriers from biopolymers present on the GS surface. Note that no spontaneous fusion events were observed in Linker-functionalized, nor plain GS–GV samples.

## 3 Conclusion

In summary, we demonstrated the electrofusion of giant spheroplasts derived from *E. coli* and giant lipid vesicles. We used both a standard approach, in which cell/vesicle proximity is non-specifically induced through an AC field, and by introducing membrane tethered DNA-constructs to selectively connect GSs and GVs, before fusion is induced by a DC pulse. We dub the latter approach DNA-assisted selective electrofusion, or DASE, and proved that, compared to non-selective (standard) electrofusion, it affords a ∼4× improvement in fusion selectivity and a ∼2× improvement in throughput. We tested two different designs for the DNA constructs: Linkers that induce adhesion between GSs and GVs, but are expected to prevent close contact between the membranes, and Zippers, designed to bring the two bilayers as close as possible. The latter perform better in terms of selectivity, and, rarely, can induce spontaneous GS–GV fusion, without the application of an electric field.

As a proof-of-concept for the applicability of DASE to intra-cellular cargo-delivery, we demonstrated the fusion of *E. coli* GSs with GVs loaded with DNA intercalating dye YOYO-1.

DASE combines the selectivity of DNA-mediated membrane fusion^60,72,73^ with the efficiency of electrofusion, and even improves on the latter, hence presenting itself as a candidate platform for all those applications requiring the high throughput delivery of large-volume payloads to biological cells *in vivo*.

A notable example is the creation of hybrid cells in the context of synthetic biology, in which both the cytoplasm and membrane composition of biological cells are altered by the fusion with a large, cargo-loaded liposome, as we demonstrated here in a simplified form. DASE could indeed be implemented to introduce, in one go, new organelles (natural or synthetic^24,25,81–84^), genetic material,^1,3^ or membrane proteins,^85,86^ all of which can be easily encapsulated in the GVs.

In addition, DASE could aid the delivery of gene-editing CRISPR machinery to cells *ex vivo*, a process required for next-generation cell-based therapeutics and currently hindered by inefficient transfection technologies.^87–89^

One limitation of the present implementation of DASE is that, because the initial DNA-mediated adhesion between GVs and cells is simply induced by bulk mixing, multimers are likely to form and lead to fusion events that involve more than a single GV and a single cell. In applications where one-to-one fusion is required, DASE may benefit from simple microfluidic traps to guarantee that only GV–cell heterodimers can form prior to applying the electric pulse.^51^ A simpler solution would be to reduce overall concentration of cells and vesicles, so to promote formation of small aggregates. However, this scenario would lead to a reduction in throughput. Additional challenges may arise from the need to pre-functionalize cells with DNA constructs. While GSs are unlikely to retain active endocytosis pathways, other cell types are known to uptake cholesterolized nanostructures,^90^ which would limit the time window available for completing DASE after first exposing the cells to the DNA constructs. Should similar issues arise, one could explore different strategies to functionalize the cells, for instance relying of different lipid anchors,^65,91^ nanobodies^92,93^ or aptamers.^94^

## 4 Methods

### 4.1 Medium, strains and inoculation

LB medium with Miller's composition was used throughout this study. The medium was prepared at 2× concentration from 20 g L^−1^ BactoTryptone (Difco Laboratories), 10 g L^−1^ yeast extract (Difco Laboratories) and 20 g L^−1^ NaCl. The medium was sterilized by filtration using PES membranes with 0.22 μm pores.

*Escherichia coli* HST08 premium competent cells were purchased from Takara Bio Inc., Japan. This strain was used to create GSs for all electrofusion experiments in the study. Upon receiving them, the competent cells were stored at −80 °C until use. The frozen cells were thawed on ice, inoculated in 3 mL of LB medium (1000× dilution), and incubated at 37 °C while shaking at 250 rpm for 2 hours. Then, the cells were inoculated on an LB agar plate and further incubated overnight. The following morning, the plate was collected and stored at 4 °C until use, which occurred within two months. After this period the plates were discarded to prevent contamination, and made anew from the stock of frozen cells.

### 4.2 Formation of *E. coli* giant spheroplast

For the preparation of *E. coli* GSs, a custom protocol was adapted from the one proposed by Kuroda *et al.*^95^ In short, *E. coli* HST08 were inoculated from an LB agar plate into 3 mL of fresh LB (Miller) medium in a 14 mL Falcon tube, and left in a shaking incubator (30/37 °C, 250 rpm) overnight. Afterwards, 50 μl of the overnight culture were added to 50 mL of fresh LB medium in a 100 mL Falcon tube (1000× dilution). The diluted culture was further incubated at 30/37 °C while shaking at 250/300 rpm. When the OD_600_ reached ∼0.25–0.75, 50 mL of a 2 M glucose solution were added to induce plasmolysis, along with 500 μl of lysozyme solution (200 μg mL^−1^) for digesting the peptidoglycan, and 500 μl of ethylenediaminetetraacetic acid (EDTA) solution (500 mM) for destabilizing the outer the membrane. The tube was then incubated at RT for 15–20 min before adding 250 μL of 1 M MgCl_2_ solution to saturate the EDTA and stop its activity. The entire lysate solution (∼100 mL) was aliquoted into two 50 mL Falcon tubes, and the aliquots were spun-down by centrifugation at 3000*g* for 5 min. The supernatant solution was discarded by inverting the Falcon tube gently. 50 mL of LB medium with 1% (w/w) bovine serum albumin (BSA) and 400 μg mL^−1^ penicillin G were then added to each Falcon tube with a lysate (spheroplast) pellet. While BSA helps avoiding adhesion of the cells to the tube surfaces, penicillin G prevents the regeneration of the peptidoglycan. Pellets of the lysates were re-dispersed by inverting, shaking and vortexing. The dispersed lysates were then incubated at 30/37 °C overnight, without shaking. Afterwards, formed GSs were collected by centrifuging at 3000–6000*g* for 5–20 min at RT, discarding the supernatant solution and adding 1 mL of the supernatant back in the tube.

### 4.3 Fluorescent staining of *E. coli*

FM4-64, SYTO41, and Wheat Germ Agglutinin-conjugated Alexa Fluor 488 (WGA–AF488) were purchased from Thermo Fisher Scientific. Native *E. coli* (HST08), spheroplasts, and GSs were stained in a given medium (typically LB medium with 1% BSA and 400 μg mL^−1^ penicillin G) by adding the dyes to the required concentrations. To stain the cytoplasmic membrane, FM4-64 (1 mg mL^−1^ stock in DMSO) was added at 10 μg mL^−1^. To stain the nucleoid, SYTO41 (5 mM in DMSO) was added at 5 μM. To stain the peptidoglycan cell wall, Wheat Germ Agglutinin, Alexa Fluor 488 Conjugate (WGA–AF488; 1 mg mL^−1^ in water) was added at 10 μg mL^−1^.^96^ The stained cells were directly used in experiments without washing.

### 4.4 Electroformation of giant lipid vesicles

Giant lipid vesicles (GVs) were prepared by standard electroformation.^72,97,98^

1,2-Dioleoyl-*sn-glycero*-3-phosphocholine (DOPC) and TopFluor Cholesterol (TFC) were purchased from Avanti Polar Lipids Inc. ATTO390-DPPE was purchased from ATTO-TEC GmbH. Stock solutions in chloroform were prepared at 100 mM for DOPC and 0.5 mM for TFC and ATTO390-DPPE, and stored at −30 °C. YOYO-1 (1 mM stock in DMSO) was purchased from Thermo Fisher Scientific.

100 μL of the 100 mM DOPC stock solution were mixed with 400 μL of one of the 0.5 mM stock solutions of fluorescent probes (TFC or ATTO390-DPPE) in a clean glass bottle, resulting in a DOPC/probe molar ratio of 98/2. The lipid solution was then spin-coated onto the conductive side of two 5 × 5 cm^2^ indium tin oxide (ITO)-coated glass slides, by spinning at 400 rpm for 180 s. A U-shaped spacer cut from a silicone rubber sheet (thickness 2 mm) was sandwiched between the two lipid-coated ITO slides, and secured with metal clips. The so-formed chambers was filled with a 340 mM sucrose solution, with or without 5 μM YOYO-1.

The slides were connected to a function generator using metal tape, and a rectangular alternating field with a frequency 10 Hz an amplitude of 4 V_p–p_, 50% duty cycle, and no offset was applied for 2 hours. Then the frequency was reduced to 2 Hz, for further 2 hours.

At the end of this process, the resulting GVs were collected with a disposable plastic pipette and dispersed into an iso-osmolar solution of 140 mM glucose with 100 mM NaCl. The GVs were then rinsed with the same iso-osmolar solution at least once by centrifuging at 1000*g* for 5 min at RT, removing the supernatant, and resuspending.

### 4.5 Assembly of DNA constructs

The sequences of all single stranded DNA components of Linkers and Zippers are reported in Table S1.†

All oligonucleotides were purchased from Eurogentec (Belgium) and received as lyophilized powder. The oligonucleotides were reconstituted in TE buffer (10 mM Tris, 1 mM EDTA) with added 100 mM NaCl to achieve a DNA concentration of 100 μM. The resulting stock solution was splitted into aliquots of ∼100 μL and stored at −30 °C until use.

Hybridization of the constructs was carried out by diluting the 8 μL of the required ssDNA components into 109 μL of TE + 100 mM NaCl buffer, then splitting the solution into 3 PCR tubes and annealing them on a thermal cycler (Veriti, Thermo Fisher Scientific) by first holding at 80 °C for 5 min and then cooling to 4 °C over about 18 hours. The assembled constructs were stored at 4 °C until use.

### 4.6 Functionalization of GSs and GVs

The functionalization of GSs with cholesterolized DNA constructs (Linkers and Zippers) was performed as follows. GSs in 50 mL of a solution of LB medium with 1% BSA and 400 μg mL^−1^ penicillin G, were spun-down at 3000/6000*g* and RT for 10–30 min. The supernatant solution was gently discarded by inverting the tube, and 920 μL of the removed solution were added back. Then, 80 μL of a solution containing the previously assembled constructs (Linkers or Zippers) at a concentration of 6.4 μM in TE buffer and 100 mM NaCl were added. This sample was mixed by tapping and vortexing, and incubated at RT for 2 hours. Afterwards, 50 mL of LB medium with 1% BSA and 400 μg mL^−1^ penicillin G were added, and the solution was mixed by inverting the tube several times. The GS solution was then centrifuged at 3000/6000*g* for 5–20 min and the supernatant was discarded by inverting the tube gently. Finally, the functionalized GS pellet was dispersed into 1 mL of LB medium with 2% BSA and 800 μg mL^−1^ penicillin G.

The functionalization of GVs was carried out as follows. Into a 1.5 mL Eppendorf tube we mixed 60 μL of a solution containing 100 mM NaCl + 226 mM glucose and 40 μL of the previously annealed solution of one of the DNA constructs (6.4 μM in 100 mM NaCl with 10 mM Tris and 1 mM EDTA). Then, 100 μL of a dense solution of electroformed GVs in 140 mM glucose with 100 mM NaCl was added. The osmolarities of the DNA-containing and the GV containing solutions were kept equal (340 mOsm) to prevent osmotic shock of the vesicles. The GV–DNA mixture was then mixed with a pipette, centrifuged at 2000*g* for 3 s and incubated at RT overnight (>18 hours). Afterwards, the sample was washed by filling the tube with a solution of 140 mM glucose with 100 mM NaCl, centrifuging at 1000*g* for 5 min, and gently discarding the supernatant by pipetting. The washing was repeated at least once. After removing the supernatant, 200 μL of the 140 mM glucose + 100 mM NaCl solution were added to the GV pellet, which was re-dispersed by tapping.

### 4.7 Fabrication of custom electrofusion devices

Copper-foil tape, 10 mm wide (Teraoka Seisakusho Co., Ltd, Japan), was cut into segments with a length of 50 mm. Two of these segments were attached onto a glass coverslip (thickness No. 1, 30 × 40 mm^2^; Matsunami Glass Ind. Ltd, Japan), parallel to each other and separated by a 0.7 mm gap. Double-sided sticky tape, 10 mm wide, was cut into a length of 20 mm and attached onto each of the two copper-tape segments, leaving the gap between them uncovered. Finally, a second No. 1 coverslip, 20 × 20 mm^2^, was adhered onto the sticky tape. The result is a narrow chamber with a rectangular cross section enclosed between the two edges of the copper-foil tapes and the two coverslips, with both ends left open. If required, additional pieces of copper tape were used to extend the electrodes. See Fig. S10† for a picture of the device.

### 4.8 Electrofusion

For both NSE and DASE, samples containing GSs (in LB medium with 2% BSA and 800 μg mL^−1^ penicillin G) and GVs (in 140 mM glucose with 100 mM NaCl) were mixed in equal volume. If the GV sample was found to be too dense, it was diluted beforehand. For DASE, the mixed GS–GV sample was incubated for 2 hours at RT prior to electrofusion.

Electrofusion was performed by loading about 10 μL of the sample of interest into a tailor made microscopy chamber equipped with two copper electrodes, shown in Fig. S10.† The device was placed on the microscope stage and connected to a voltage supply (LF101 Cell Fusion Unit, BEX CO., Ltd, Japan).

For NSE, an alternating field with amplitude of 4 V_RMS_ and frequency of 1.9 MHz was applied while monitoring the sample, until sufficient chain-like aggregates of GVs and GSs were formed. The duration of this phase changed from sample to sample. After aggregate formation, a DC pulse with an amplitude of 600 V mm^−1^ and a duration of 50 μs was applied to induce the fusion.

For DASE, the previously incubated GS–GV samples were loaded in the electrofusion device and the DC pulse was applied without previously applying the AC field.

For both NSE and DASE, the fusion process was monitored by recording at 2.9 fps in random locations while imaging with the 60× objective, and the so-collected data were used for the quantitative assessment of fusion selectivity, density and probability (Fig. 7, S6 and Tables S2–S5†). Alternatively, when imaging with higher magnification objective (100×), fields of view were selected manually to maximize the number of GS–GV interacting pairs. Data from non-randomly-selected fields of view were excluded from statistical analysis to avoid bias. Images shown in Fig. 8 and S7, S8, ESI Videos 5, 8 and 12† were collected with the 60× objective, while images shown in Fig. 1C, E, H, J, 2B, 4A, 5, 6 and S3–S5, S9, ESI Videos 1–4, 6, 7 and 9–11† were collected with the 100× objective. See Microscopy section (Methods) for technical details.

### 4.9 Microscopy

All microscopy data were collected on an inverted microscope (IX71, Olympus, Japan) equipped with a CSU-X1 spinning disk confocal unit (Yokogawa, Japan), an EM-CCD (iXon X3, Andor Technology, UK), a laser combiner (ALC5000, Andor Technology, UK), and a Piezo motorized stage (Prior Scientific). All imaging sequences were programmed and performed with an iQ2 software (Andor Technology). Imaging was carried out either with a PlanApoN 60× 1.45 NA oil immersion objective (field of view 113 × 113 μm^2^) or with a UPlanSApo 100× 1.40 NA oil immersion objective lens (field of view 67 × 67 μm^2^), both from Olympus. Laser illumination was provided by one of three diode lasers in the Andor laser combiner through a triple band pass dichroic beamsplitter (405/488/561 nm Yokogawa dichroic beamsplitter, Semrock). For fluorescence detection, one of three single band bandpass filters (460/80 nm, 520/35 nm and 617/73 nm BrightLine single-band bandpass filters, Semrock) was chosen from iQ2 software.

### 4.10 Pair correlation function

The pair correlation function (radial distribution, *g*(*r*)) between GSs and GVs was computed using a tailor made MATLAB script according to the following procedure for each combination of the DNA functionalizations of GSs and GVs (Fig. 4B–D and Fig. S2†). First, the script located circles in the SYTO41 and TopFluor cholesterol channels, corresponding to the nucleoid of GSs and the membrane of GVs, respectively. The centers of mass of both object types were then calculated and used to compute the GS–GV radial pair correlation function as1
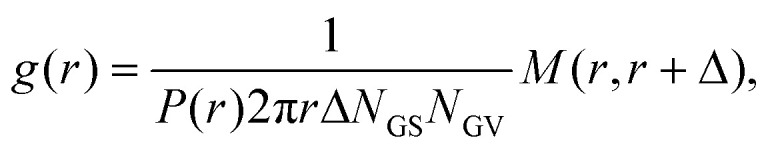
where *N*_GS_ and *N*_GV_ are respectively the numbers of localized GSs and GVs in the field of view, and *M*(*r*, *r* + *Δ*) is the number of GS–GV pairs whose centers of mass are separated by a distance between *r* and *r* + *Δ*. The bin size *Δ* was set to 3.3 μm. *P*(*r*) is a normalization factor, computed as the pair distribution function of two groups of *N*_R_ = 512 randomly picked points in a box of the same size and shape as the microscopy image2
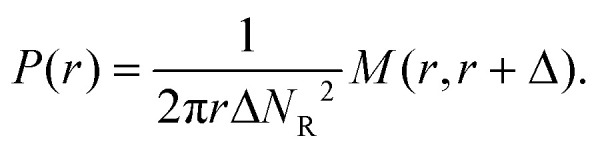


### 4.11 Fusion selectivity, density and probability

Fusion selectivity (*σ*, Fig. 7B), areal density (*ρ*, Fig. 7C), and probability (Fig. S6B–D†), were computed from the numbers of fusion events (GS–GV, GS–GS and GV–GV), GSs and GVs were counted in each microscopy field of view during an NSE/DASE run, and summarized in Tables S2–S5 and Fig. S6A.† GSs, GVs, and fusion events were identified and classified by manual inspection of the confocal movies. The first frame of each movie was used to count GSs and GVs (Tables S2–S5†). Confocal videos were recorded with a 60× objective lens at random locations in the samples (see Microscopy section in Methods). *P*-Values were computed by performing multiple testing using MATLAB multcompare function, making Bonferroni corrections of significance level (*α* = 0.05), and presented partially using conventional asterisk annotation (*: *p* ≤ 0.05, **: *p* ≤ 0.01, ***: *p* ≤ 0.001).

### 4.12 Radial distribution of FM4-64, TFC, WGA–A488, and SYTO41 fluorescence

The radial fluorescence distribution of FM4-64, TopFluor Cholesterol (TFC), Wheat Germ Agglutinin-conjugated Alexa Fluor 488 (WGA–AF488) and SYTO41 signals from native *E. coli*, spheroplast, GS, TFC-labelled GV and fused cells (see Fig. S1 and S9†), were computed with the following procedure. When present (Fig. S1A, C and S9A, C†), the center of mass (CM) of the native *E. coli*, spheroplast, GS or hybrid cell, was located as that of the SYTO41 signal, computed with Gaussian filtering and thresholding. For GVs we calculated instead the CM from the TFC signal. Using the computed CM as a reference point, the radial profiles of the three fluorescent channels were computed along 360 uniformly distributed radial directions. The angular average of these profiles is shown in Fig. S9.† Data in Fig. S1† were obtained by further averaging over multiple cells of each type.

## Author contributions

L. Di Michele, S. Takeuchi and P. Cicuta conceived the project. S. Takamori performed all experiments and data analysis. All authors participated in the interpretation of data, manuscript writing and the revisions. All authors approved the final version of the manuscript.

## Conflicts of interest

There are no conflicts to declare.

## Supplementary Material

NR-014-D2NR03105A-s001

NR-014-D2NR03105A-s002

NR-014-D2NR03105A-s003

NR-014-D2NR03105A-s004

NR-014-D2NR03105A-s005

NR-014-D2NR03105A-s006

NR-014-D2NR03105A-s007

NR-014-D2NR03105A-s008

NR-014-D2NR03105A-s009

NR-014-D2NR03105A-s010

NR-014-D2NR03105A-s011

NR-014-D2NR03105A-s012

NR-014-D2NR03105A-s013
