# Supplementary material for: DNA-assisted selective electrofusion (DASE) of Escherichia coli and giant lipid vesicles
Source: Nanoscale. 2022 Aug 24;14(38):14255–67. doi: 10.1039/d2nr03105a (PMC9536516; doi:10.1039/d2nr03105a)
Supplement: NR-014-D2NR03105A-s001 [file NR-014-D2NR03105A-s001.pdf]

Electronic Supplementary Material (ESI) for Nanoscale.
This journal is © The Royal Society of Chemistry 2022

Electronic Supplementary Information: DNA-Assisted Selective Electrofusion (DASE) of *Escherichia coli* and Giant Lipid Vesicles

Sho Takamori,[†,‡,¶] Pietro Cicuta,[*,†] Shoji Takeuchi,[*,‡,§,‖,¶] and Lorenzo Di Michele[*,⊥,#,†]

†*Department of Physics, University of Cambridge, United Kingdom*

‡*Institute of Industrial Science, the University of Tokyo, Japan*

¶*Artificial Cell Membrane Systems Group, Kanagawa Institute of Industrial Science and Technology, Japan*

§*Department of Mechano-Informatics, Graduate School of Information Science and Technology, the University of Tokyo, Japan*

‖*International Research Center for Neurointelligence (IRCN), the University of Tokyo Institutes for Advanced Study (UTIAS), the University of Tokyo, Japan*

⊥*Department of Chemistry, Imperial College London, United Kingdom*

#*fabriCELL, Imperial College London, United Kingdom*

E-mail: pc245@cam.ac.uk; takeuchi@hybrid.t.u-tokyo.ac.jp; l.di-michele@imperial.ac.uk

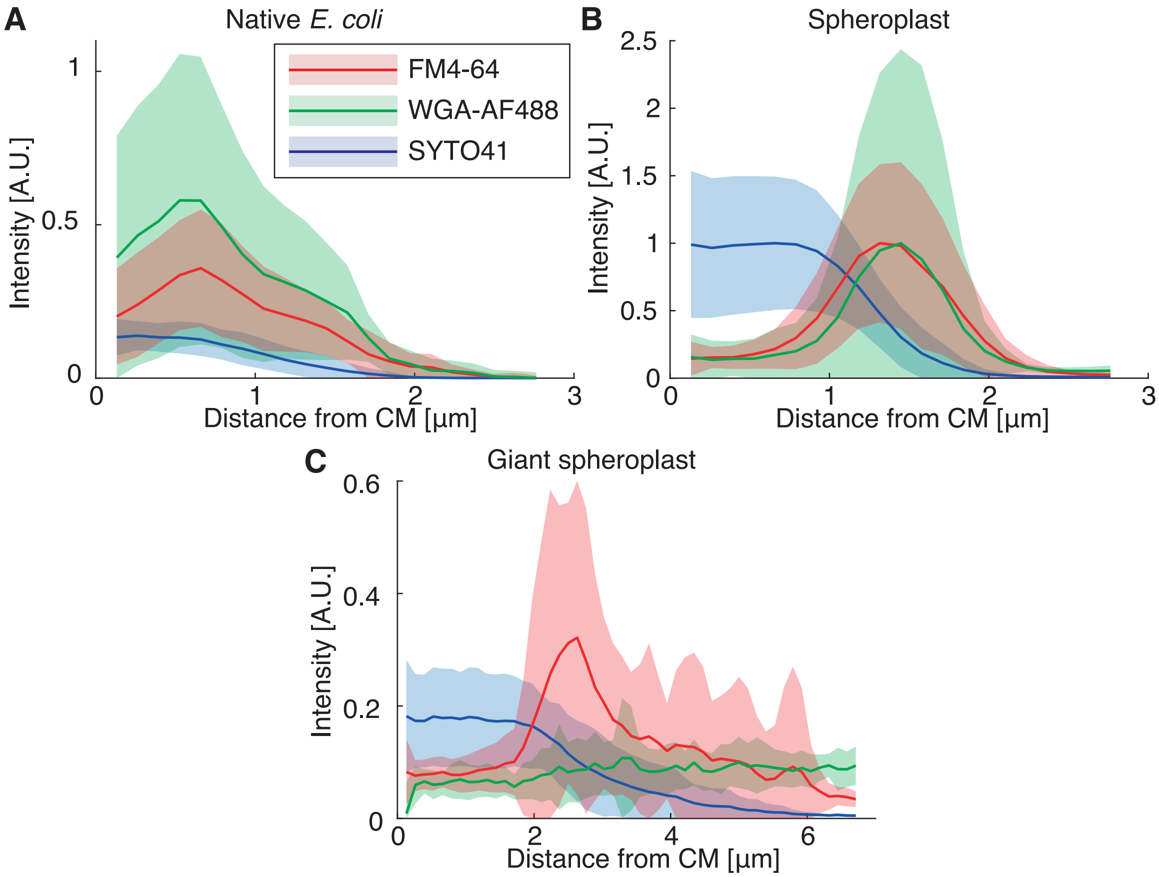

Figure S1: ***Radial distribution of the fluorescent emission of the dyes staining the cytoplasmic membrane (FM4-64), peptidoglycan (WGA-AF488), and nucleoid (SYTO41) for native E. coli, spheroplasts and GSs.*** (A) Native *E. coli.* (B) spheroplast. (C) giant spheroplast. For each, the center-of-mass (CM) of the SYTO41 signal was computed, and the radial intensity profile from the CM was extracted at 360 uniformly distributed angles in the FM4-64, WGA-AF488 and SYTO41 channels. The extracted radial intensity was averaged over all angles. A population average was computed over $N = 10$ cells for native *E. coli*, $N = 11$ cells for spheroplasts, and $N = 12$ cells for GSs. The mean over the cell populations is indicated by a solid line, while the shaded region indicates one standard deviation. A shift of the FM4-64 membrane peak towards larger radial distances is observed as the cells grow from native *E. coli* to GSs. Accordingly, the SYTO41 nucleoid distribution becomes broader. The WGA-AF488 (peptidoglycan) peak is evident in native *E. coli* and spheroplasts, indicating that some peptidoglycan is left on the spherplasts despite the digestive action of lysozyme. The WGA-AF488 disappears in GSs, indicating that the peptidoglycan detaches from the membrane as the spheroplats grow. Compare with confocal micrographs in Fig. 2B.

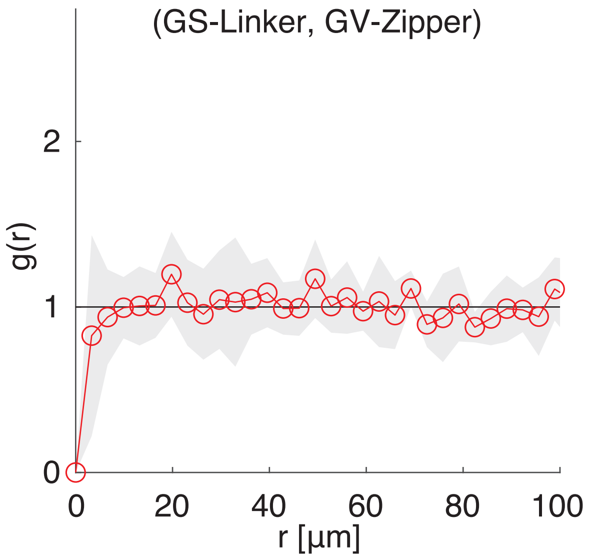

Figure S2: ***Radial pair-distribution functions*** $g(r)$ ***of GSs functionalized with Linker constructs and GVs functionalized with Zipper constructs.*** The absence of a maximum at short separations suggests the lack of GS-GV adhesion, as expected since GSs and GVs are decorated with non-complementary DNA constructs. Compare with Fig. 4B-D.

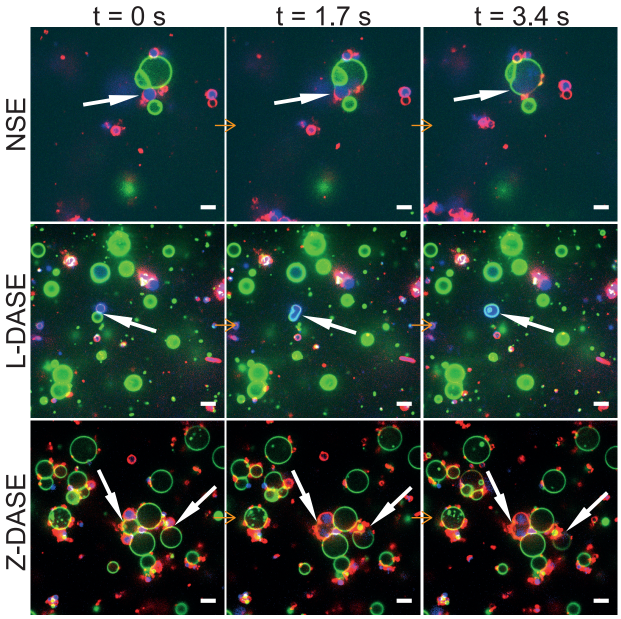

Figure S3: ***Confocal microscopy demonstrates Non-Selective and DNA-Assisted Selective Electrofusion of GSs and GVs.*** In NSE, an AC field (1.9 MHz and $4\,V_{RMS}$) was applied leading the formation of non-specific chain-like aggregates of GSs and GVs. For L-DASE and Z-DASE, GSs and GVs were mixed and incubated for 2 hours prior to the experiment. The formation of adhering GS-GV pairs is visible. At $t = 0$ s, a DC pulse (50 µs width and $600\,V\,mm^{-1}$) was applied to induce fusion, which in all cases occurred by $t = 1.7$ s. By $t = 3.4$ s, spreading of the GS nucleoid into the cytoplasm of the newly formed hybrid cell is visible, as is the mixing of the GS and GV membranes. Relaxation of the fused membranes into more spherical shapes is also visible by comparing frames at $t = 1.7$ s and $t = 3.4$ s. GSs were stained with FM4-64 (red, cytoplasmic membrane) and SYTO41 (blue, nucleoid). GVs were labelled with TopFluor Cholesterol (TFC, green, DOPC/TFC = 98/2 molar ratio). Scale bars: 5 µm. Movies of the events are shown in Supplementary Videos 3 (NSE), 7 (L-DASE) and 11 (Z-DASE).

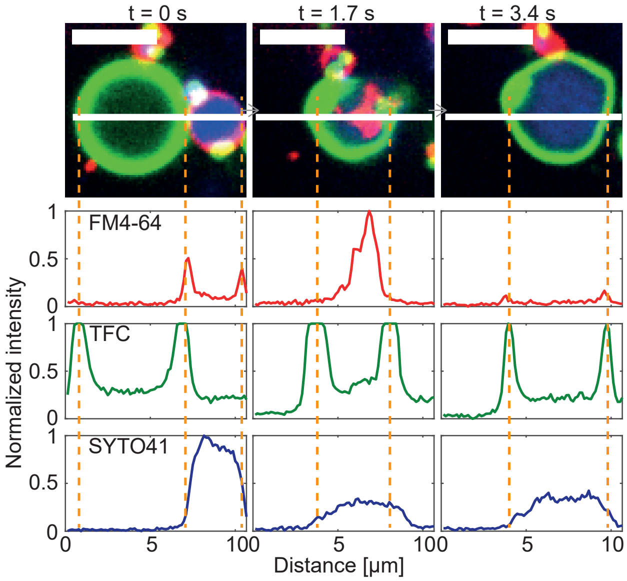

Figure S4: ***Cross section-based confirmation of membrane fusion between GSs and GVs in an L-DASE experiment.*** Three consecutive confocal microscopy frames of an L-DASE event are shown (top), where the membrane and nucleoid of GS are shown in red (FM4-64) and blue (SYTO41), respectively, and the GV membrane is shown in green (TopFluor Cholesterol/TFC). A DC pulse is applied just after $t = 0\,\mathrm{s}$ ($50\,\mu\mathrm{s}$ duration, $600\,\mathrm{V\,mm^{-1}}$ amplitude). Fluorescence intensity cross-sections for the three channels were extracted along the white line. Scale bars: $5\,\mu\mathrm{m}$. See Supplementary Video 6.

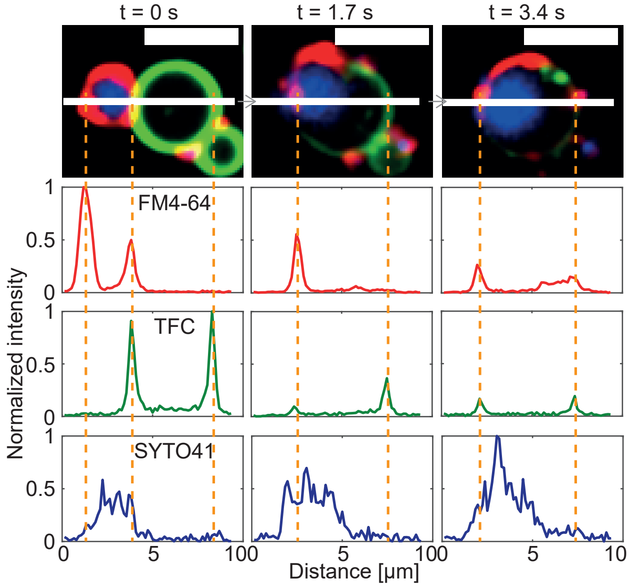

Figure S5: ***Cross section-based confirmation of membrane fusion between GSs and GVs in a Z-DASE experiment.*** The same description as in the caption of Fig. S4 applies here, but Zippers were used instead of Linkers. The movie of the event is shown in Supplementary Video 10.

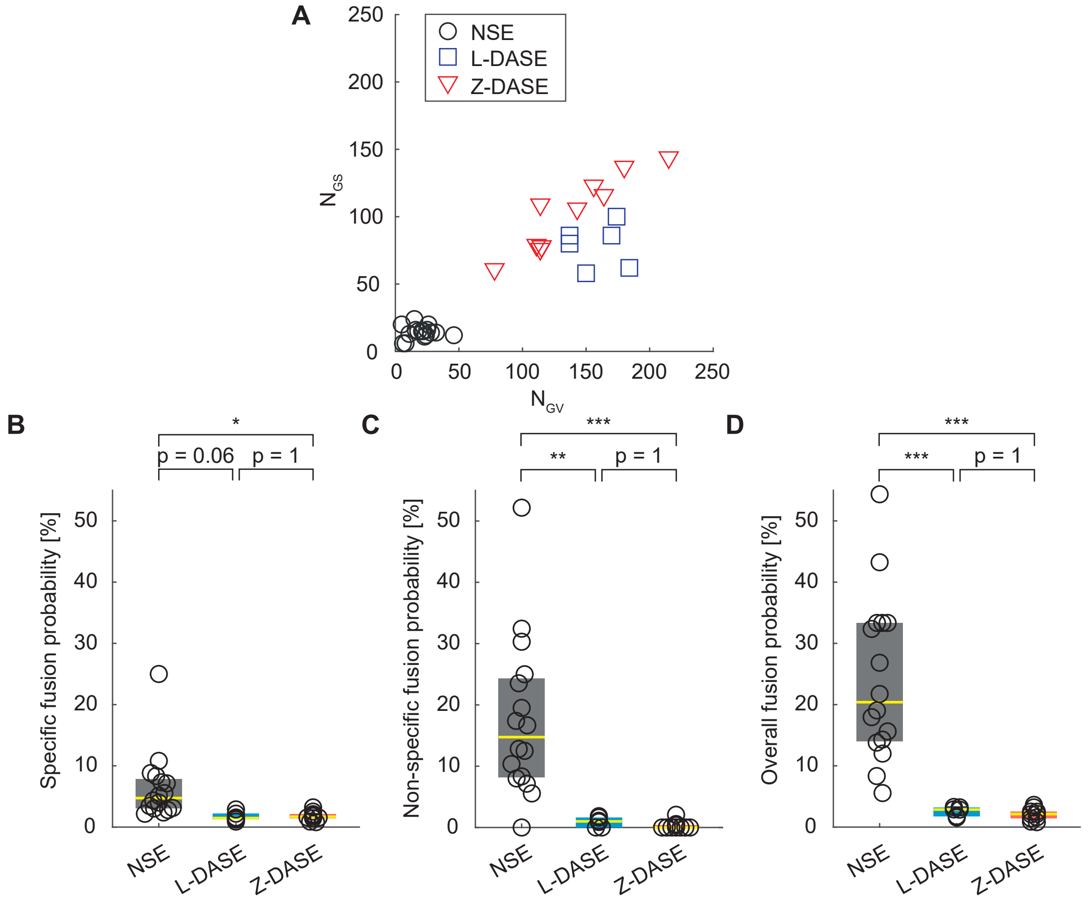

Figure S6: ***Total number of GVs (N_{GV}) and GSs (N_{GS}) and specific/non-specific/overall fusion probability observed in electrofusion experiments.*** (A) Scatter plot of N_{GS} vs N_{GV} for NSE, L-DASE and Z-DASE. The computed averages are, $N_{\mathrm{GV}} = 20 \pm 11$ and $N_{\mathrm{GS}} = 14 \pm 5$ for NSE, $N_{\mathrm{GV}} = 159 \pm 20$ and $N_{\mathrm{GS}} = 79 \pm 16$ for L-DASE, and $N_{\mathrm{GV}} = 139 \pm 40$ and $N_{\mathrm{GS}} = 103 \pm 28$ for Z-DASE (Mean ± SD). See Methods in the main text for the counting procedure and Table S5 for the raw data. (B) The specific fusion probability is defined by the number of GS-GV fusion events divided by the total number of GSs and GVs in a field of view. The computed median is 4.7% for NSE, 1.5% for L-DASE and 1.7% for Z-DASE. (C) The non-specific fusion probability is defined by the number of GV-GV and GS-GS fusion events divided by the total number of GSs and GVs. The computed median is 14.7% for NSE, 1.0% for L-DASE and 0% for Z-DASE. (D) The overall fusion probability is defined by the number of all fusion events (GS-GV+GV-GV+GS-GS) divided by the total number of GSs and GVs. The computed median is 20.4% for NSE, 2.9% for L-DASE and 2.1% for Z-DASE. Each boxplot in panels B-D is drawn with a median (yellow line) and 25-75% centile (dark grey/blue/red rectangle). See Methods in the main text for the computation and annotation of p-values.

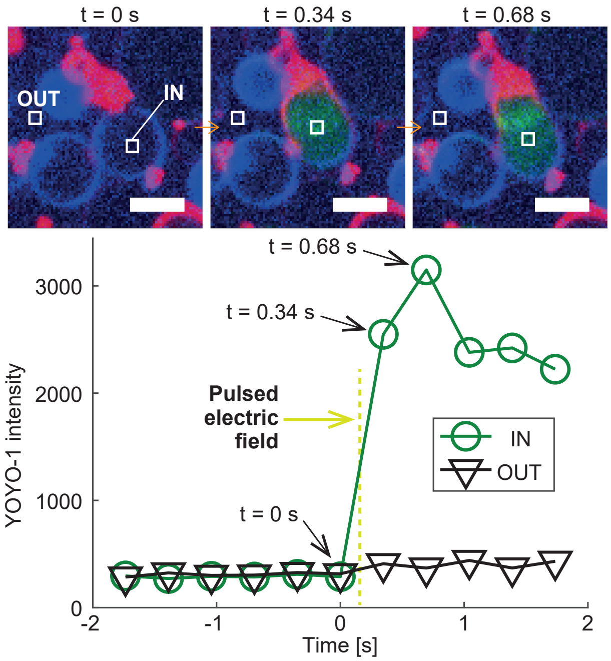

Figure S7: ***Intracellular delivery of GV-encapsulated YOYO-1 into a GS via fusion between GSs and GVs in an L-DASE experiment.*** The DNA-intercalating dye YOYO-1 was encapsulated in the GVs, whose membranes were stained with ATTO390-DPPE (blue). GSs were only labelled with the membrane stain FM4-64 (red). Prior to the electrofusion experiment, both GSs and GVs were functionalized with Linkers and mixed to enable adhesion as described in Methods in the main text. The YOYO-1 signal (green) was monitored in a region of interest inside the GV (IN) and one in a nearby empty region (OUT), and the average intensity recorded in each region is shown as a function of time (bottom). The event is shown in Supplementary Video 8. See discussion in the main text and Fig. 8, showing analogous trends from a Z-DASE experiment. Scale bars: 5 µm.

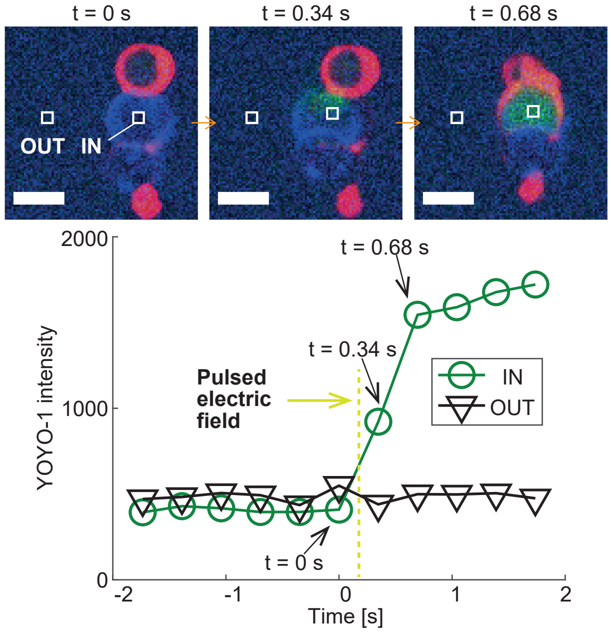

Figure S8: ***Intracellular delivery of vesicle-encapsulated YOYO-1 into a GS via fusion between GSs and GVs in an NSE experiment.*** The same description as in the caption of Fig. S7 applies here except that GSs and GVs were not functionalized with DNA and AC field was applied for the formation of the chain-like aggregate prior to the acquisition of images. The event is shown in Supplementary Video 5. See discussion in the main text and Fig. 8, showing analogous trends from a Z-DASE experiment. Scale bars: 5 µm.

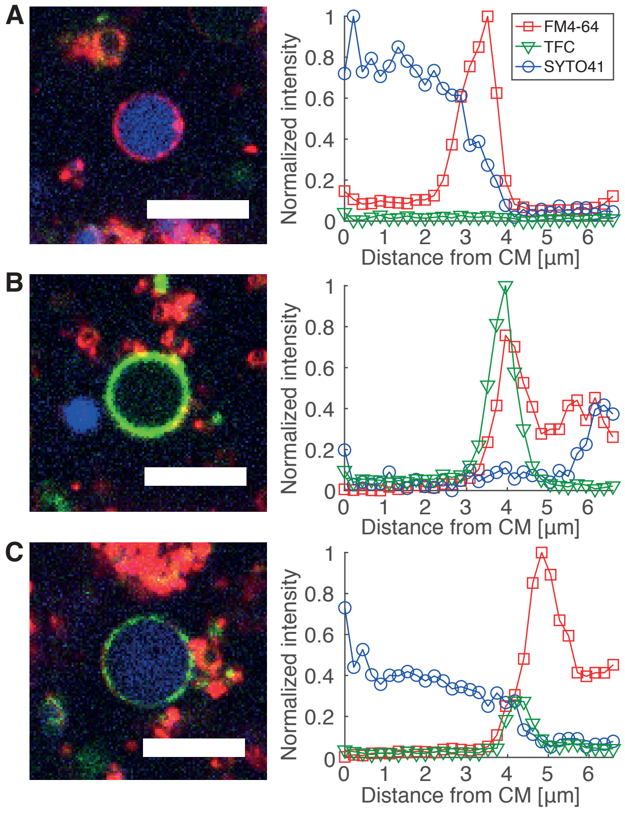

Figure S9: ***Zippers can mediate spontaneous GS-GV fusion***. Zipper decorated GSs and GVs were mixed and incubated for 18 hours before confocal imaging. The cytoplasmic membrane of GSs was stained with FM4-64 (red) and their nucleoid with and SYTO41 (blue). GVs were labelled by TopFluor Cholesterol (TFC, green). Panels A-C show confocal micrographs of a typical GS (A), a typical GV (B) and a hybrid cell formed after DNA-mediated fusion (C). The radially averaged fluorescence of the three different channels, shown as a function of the distance from the cell's center of mass (CM, see Methods in the main text), demonstrates the occurrence of membrane fusion and content mixing. Scale bars: 10 µm.

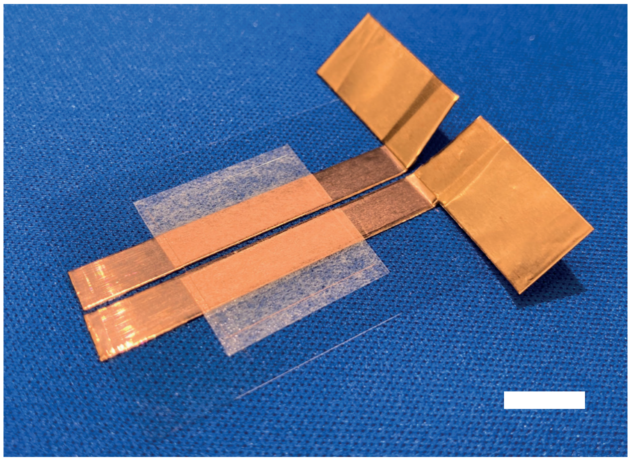

Figure S10: ***Custom-made electrofusion device.*** See Methods in the main text for details on fabrication. Scale bar: 10 mm.

Table S1: ***Sequences of the single-stranded components of Linkers and Zippers.*** Non-fluorescent Linker with sticky end α and its complementary with sticky end α^* were assembled by annealing ssLinker1 with ssLinker2 and ssLinker3, respectively. In the fluorescent versions used in Fig. 4A, ssLinker2 was replaced with ssLinker4 and ssLinker3 with ssLinker5. Zippers with ssDNA domains β, γ, θ and ϕ were assembled by annealing ssZipper1 with ssZipper2, while Zippers with the corresponding complementary domains using ssZipper3 with ssZipper4. ssDNA domains α/α^* are shown in red, β/β^* in orange, $\gamma/\gamma*$ in blue, θ/θ^* in purple, ϕ/ϕ^* in green and δ/δ^* in cyan. Compare with Fig. 3 and see Methods in the main text for a description of the assembly protocol.

Name	Sequence (5'→3')
**ssLinker1**	Cholesteryl-TEG-TTCCTGCCTTACGATGC
**ssLinker2**	TTCGATGGTAGCTTGCATCGTAAGGCAGGTT-TEG-Cholesteryl
**ssLinker3**	TTGCTACCATCGTTGCATCGTAAGGCAGGTT-TEG-Cholesteryl
**ssLinker4**	ATTO488-TTCGATGGTAGCTTGCATCGTAAGGCAGGTT-TEG-Chol esteryl
**ssLinker5**	ATTO590-TTGCTACCATCGTTGCATCGTAAGGCAGGTT-TEG-Chole steryl
**ssZipper1**	GCCACTTCACCCGACCATTCTGGCCGTTGCGCTCGTGAAAGTAGC -TEG-Cholesteryl
**ssZipper2**	Cholesteryl-TEG-CGAGTAAGGGCGAGCGCAACGGCCAGAATGGTC GGTCACTCAAAC
**ssZipper3**	Cholesteryl-TEG-GCTACTTTCACGAGCGCAACGGCCAGAATGGTC GGGTGAAGTGGC
**ssZipper4**	GTTTGAGTGACCGACCATTCTGGCCGTTGCGCTCGCCCTTACTCG -TEG-Cholesteryl

Table S2: ***The number of observed GS-GV, GV-GV and GS-GS fusion events in NSE experiments (*** $N = 16$ ***).*** See Methods in the main text for experimental and data analysis information.

GS-GV	GV-GV	GS-GS
1	24	0
2	5	1
3	8	0
2	6	2
1	10	0
4	12	0
2	3	2
1	1	1
2	4	2
3	1	0
3	8	0
1	5	2
1	1	0
1	2	0
1	4	0
2	0	0

Table S3: ***The number of observed GS-GV, GV-GV and GS-GS fusion events in L-DASE experiments ($N = 6$).*** See Methods in the main text for experimental and data analysis information.

GS-GV	GV-GV	GS-GS
6	0	0
3	4	0
6	3	0
2	2	0
4	0	0
3	4	0

Table S4: ***The number of observed GS-GV, GV-GV and GS-GS fusion events in Z-DASE experiments (N = 10).*** See Methods in the main text for experimental and data analysis information.

GS-GV	GV-GV	GS-GS
2	0	0
2	0	0
3	0	0
4	0	0
2	0	0
5	0	1
8	0	0
7	2	0
9	0	0
3	2	2

Table S5: ***Total number of GVs and GSs observed in individual fields of view in NSE*** ($N = 16$)***, L-DASE*** ($N = 6$) ***and Z-DASE*** ($N = 10$) ***experiments.*** See Methods in the main text for experimental details.

NSE		L-DASE		Z-DASE	
**GV**	**GS**	**GV**	**GS**	**GV**	**GS**
26	20	150	58	78	61
46	12	184	62	114	109
23	11	174	100	114	76
32	14	137	86	111	79
18	15	170	86	143	106
22	15	137	80	156	123
15	24			180	137
5	20			215	144
11	13			164	116
6	6			115	78
25	16				
28	14				
8	6				
21	15				
16	16				
24	12				
